# Triplex Proofman-LMTIA: A Rapid, Specific, and Sensitive Assay for Detecting Wheat, Peanut, and Soybean Allergens in Foods

**DOI:** 10.3390/foods15081340

**Published:** 2026-04-12

**Authors:** Linqing Guo, Dan Zhou, Chunmei Song, Chaoqun Wang, Duoxuan Liu, Yue Cao, Xiaodong Zhang, Bo Tian, Deguo Wang

**Affiliations:** 1 Food College, Northeast Agricultural University, Harbin 150000, China; 13199571175@163.com; 2 Key Laboratory of Biomarker Based Rapid-Detection Technology for Food Safety of Henan Province, Food and Pharmacy College, Xuchang University, Xuchang 461000, China; zdan13575030052@163.com (D.Z.); zhiya007@163.com (C.S.); 15837457816@163.com (Y.C.); zxd95@126.com (X.Z.); 3 Technology Center, Zhengzhou Customs District P. R. China, Zhengzhou 450000, China; 18203740159@139.com; 4 College of Food Science, Southwest University, Chongqing 400715, China; 13783740863@163.com

**Keywords:** wheat, soybean, peanut, allergen detection, multiple detection, ladder-shape melting temperature isothermal amplification

## Abstract

Wheat, soybean, and peanut are recognized as major food allergens, with their prevalence rising globally, necessitating rapid and reliable detection methods. A new detection approach was developed in this research, which integrates Ladder-shape Melting Temperature Isothermal Amplification (LMTIA) with Proofreading Enzyme-Mediated Probe Cleavage (Proofman) technology to enable the concurrent identification of wheat, soybean, and peanut allergens. Compared with the loop-mediated isothermal amplification (LAMP) method under the experimental conditions set in this study, this approach can reduce the false-positive results associated with LAMP, and it does not rely on sophisticated instrumentation required by technologies like mass spectrometry. The *GAG56D* (wheat), *Ara h 2.01* (peanut), and *Lectin* (soybean) genes were selected as target genes for the three allergens. Specific primers and probes were designed according to these target genes, and the reaction system was optimized. A systematic evaluation of the triplex Proofman-LMTIA method was then conducted regarding its specificity, sensitivity, limit of detection, and repeatability. Finally, the method’s practical applicability was validated using commercial products. The optimized system achieved simultaneous detection within 40 min at 61 °C, showing no cross-reactivity with common foods. The method demonstrated good sensitivity, with a sensitivity of 5 pg/μL for genomic DNA and a detection limit of 5% (*w*/*w*) in a powder matrix, along with excellent repeatability. In practical sample testing, the results were fully consistent with product label declarations, accurately identifying single and multiple allergen contaminations. The Proofman-LMTIA detection method, with its rapid, simple, sensitive, and specific characteristics, demonstrates significant potential for applications in food safety supervision.

## 1. Introduction

Food allergy is an abnormal immunological reaction provoked by particular allergenic substances. It presents with symptoms including pruritus, laryngeal oedema, and respiratory distress, potentially resulting in life-threatening complications in severe instances [1,2]. As a major public health issue, it impacts individuals of all age groups. The global incidence of food allergies has risen significantly, with prevalence among Chinese infants increasing from 7.7% in 2009 to 11.1% in 2019 [3]. Food allergies impact an estimated 26 million adults and 6 million children in the U.S. [4,5]. Wheat, soybean, and peanut are significant agricultural commodities worldwide that provide essential proteins and carbohydrates for human nutrition. In 2021, the United States passed the Food Allergy Safety, Treatment, Education, and Research Act (FASTER Act), and in 2023, sesame was officially designated as the ninth major allergen. Together with milk, eggs, fish, crustaceans, peanuts, soybeans, tree nuts, and wheat, these nine major allergens account for approximately 90% of all food allergy cases [6]. The industrialization of food has led to cross-contamination hazards during food production, storage, and transportation. Thus, even trace amounts of allergens pose significant health risks to sensitized individuals [7,8].

Food allergen detection technologies have been primarily categorized into those that identify allergenic proteins and those that utilize allergen-specific DNA [9]. Several methods are employed for protein-based detection. These include enzyme-linked immunosorbent assay (ELISA), lateral flow immunoassay (LFIA), xMAP technology, and mass spectrometry (MS) [10]. Among these, ELISA has been the predominant technique employed in food analysis. It depends on unique antigen–antibody interactions combined with enzymatic signal amplification to facilitate qualitative or quantitative assessment of target proteins [11]. LFIA has integrated immunochromatography with antibody–antigen recognition, facilitating rapid on-site screening [11]. The xMAP method employs fluorescence-encoded microspheres coated with specific capture antibodies, which form complexes with biotinylated detection antibodies and streptavidin–fluorophore reporters, enabling multiplex detection [12]. Immunoassays are often constrained by challenges in generating high-affinity antibodies and the risk of cross-reactivity with structurally analogous proteins, potentially resulting in false-positive outcomes. In contrast, mass spectrometry (MS) accurately identifies allergenic proteins using distinctive peptide markers, avoiding antibody-related limitations [13]. Nonetheless, MS relies on costly instrumentation and specialized expertise, limiting its use in field testing.

Food processing techniques such as high temperature and high pressure can denature allergenic proteins and may reduce allergenicity to a certain extent, but they can hardly completely eliminate the risk of allergy. Even trace amounts of residual allergens can trigger severe allergic reactions. At the same time, protein denaturation seriously reduces the reliability of immunoassays and often leads to false-negative results. In contrast, DNA-based methods achieve indirect allergen detection by targeting allergen-related DNA. Although DNA may also break down or degrade under extreme processing conditions, short target fragments (e.g., 100–200 bp) can usually still be effectively detected, showing higher stability than proteins [14]. Nucleic acid detection methods, including PCR, real-time quantitative PCR (RT-PCR), digital PCR (dPCR), high-resolution melting (HRM), and loop-mediated isothermal amplification (LAMP), are established as standard procedures in some countries [15]. In Germany, PCR and qPCR have been officially recognized as official analytical methods for the detection of various allergenic foods, including seven allergens such as wheat, rye, and soybean [16,17]. LAMP has been incorporated into the industry standards for entry-exit inspection and quarantine in China [18]. Conventional PCR has allowed for multiplex detection but requires gel electrophoresis for result visualization, and the need to open reaction tubes after amplification increases the risk of contamination. qPCR and dPCR have supported multiplex detection; however, the requisite equipment and consumables have been prohibitively expensive. High-resolution melting (HRM) analysis has required exceptionally precise temperature control systems and imposed stringent limitations on primer design. The LAMP technique requires complex primer design, risks aerosol contamination, and is highly prone to false positives. Consequently, a rapid, reliable, simple, and sensitive detection method is needed. Ladder-shape Melting Temperature Isothermal Amplification (LMTIA) is an innovative nucleic acid amplification technique that has been effectively utilized in species authentication [19,20,21], virus detection [22], and the identification of traditional Chinese medicines [23]. The technique is based on differences in melting temperature (Tm) between primers and target sequences, enabling the amplification of single-stranded DNA without thermal denaturation or specialized auxiliary enzymes such as helicase or recombinase. A ladder-like Tm distribution across the target is essential for reaction initiation. The procedure consists of two stages: an initial phase producing dumbbell-shaped DNA intermediates, followed by an exponential amplification phase. Both phases involve iterative annealing, strand displacement, and synthesis [24]. In contrast to conventional PCR, LMTIA is performed under isothermal conditions, removing the necessity for a thermal cycle, reducing detection time, and enhancing sensitivity and stability. Bst DNA polymerase, the key enzyme, has demonstrated significant strand-separating capability and heat resistance, enabling successful copying of target sequences down to 60 bp from ssDNA, dsDNA, or RNA templates. This method requires only a miniature isothermal amplification instrument for detection, eliminating the risk of false positives while enabling multiplex detection. Moreover, for food allergen detection specifically, such a method must not only possess these advantages but also adapt to complex food matrices, enable multiplex detection to address the frequent occurrence of multiple allergen contamination, and maintain sufficient robustness. Leveraging its isothermal nature, short amplification time, and compatibility with fluorescence-based closed-tube detection, LMTIA technology holds promise for fulfilling these specific requirements, demonstrating broad application prospects in the field of allergen detection.

Allergen DNA targets may originate from gene sequences that encode allergenic proteins or from species-specific DNA fragments [15]. Official allergy databases indicate that wheat, soybean, and peanut contain 28, 33, and 17 allergenic proteins [25]. γ-Gliadin (Tri a 20) is widely recognized as one of the major wheat allergens, involved not only in classical wheat allergy but also in wheat-dependent exercise-induced anaphylaxis and hydrolyzed wheat protein-related allergy. A study conducted in Thailand confirmed its role as a key sensitizing protein in adult-onset wheat allergy, with IgE reactivity detected in 100% of patients with classical wheat allergy [26]. Ara h 2 is the primary allergen responsible for peanut allergy, with detection of Ara h 2-specific IgE serving as a diagnostic marker for peanut allergy [27]. Two subtypes of Ara h 2 have been recognized: Ara h 2.01 and Ara h 2.02. The former is more common. It is found in more than 90% of peanut-allergic patients [28]. In soybean, due to the multitude of soybean allergenic proteins and the genetic diversity of their genes, detection methods relying on a single allergenic gene may lack reliability. Following the Chinese industry standards for entry-exit inspection and quarantine, which specify the soybean lectin gene as the target for allergen detection, this study also employed this gene to detect soybean allergens [18]. Thus, we selected the *GAG56D*, *Ara h 2.01*, and *Lectin* genes as robust targets. These allow for the specific identification of wheat, peanut, and soybean, respectively. Based on our previously established single-plex Proofman-LMTIA detection methods for soybean, wheat, and peanut, as well as the duplex Proofman-LMTIA method for soybean and wheat allergens [29], this paper establishes, for the first time, a triplex Proofman-LMTIA method for the simultaneous detection of soybean, wheat, and peanut allergens. The transition from duplex to triplex detection is not merely the addition of a third target. First, it is essential to ensure that the optimal reaction temperatures of the three allergen systems are similar. This requires optimization across multiple systems and selection of sufficiently close temperatures to guarantee the feasibility of the triplex reaction. Second, key components of the reaction system, such as the 5× premix buffer, Mg^2+^, and Bst polymerase, require optimization to ensure comparable amplification efficiency among the three targets. Additionally, rigorous validation experiments are needed to maintain high sensitivity and specificity despite the increased reaction complexity.

The Proofman-LMTIA method was first described by Wang et al. [30]. Subsequently, the duplex Proofman-LMTIA method has also been successfully applied to the detection of corn and sweet potato in starch [31], as well as the identification of Panax quinquefolius and Panax ginseng [23]. This study represents the first application of triplex Proofman-LMTIA for the simultaneous detection of three major food allergens.

## 2. Materials and Methods

### 2.1. Materials

Various food samples were acquired from the Xiyingmen Supermarket (Xuchang, China). These included rice (*Oryza sativa*), corn (*Zea mays*), soybean (*Glycine max*), peanut (*Arachis hypogaea*), wheat (*Triticum aestivum*), walnut (*Juglans regia*), sesame (*Sesamum indicum*), cashew (*Anacardium occidentale*), almond (*Prunus dulcis*), nut bread, oatmeal, and soda crackers, among others.

Several reagents and kits were obtained from different suppliers. A plant genomic DNA (gDNA) extraction kit was sourced from Tianlong Science and Technology Co., Ltd. (Xi’an, China). Bst DNA polymerase came from Shandong Med Biotechnology Co., Ltd. (Jinan, China). The 5× reaction mixture was supplied by Dege Biotechnology Co., Ltd. (Heze, China). Diethyl pyrocarbonate-treated water (DEPC-H_2_O) was purchased from Hefei Laier Bioengineering Co., Ltd. (Hefei, China), while liquid paraffin was bought from Shanghai Sangon Bioengineering Co., Ltd. (Shanghai, China). Except where noted, all other reagents were provided by China National Pharmaceutical Group Chemical Reagent Co., Ltd. (Shanghai, China).

### 2.2. Design of Primers and Probes

Sequences for the soybean *Lectin* gene, wheat *GAG56D* gene, and peanut *Ara h 2.01* gene were retrieved from the NCBI GenBank database. The analysis and screening of target sequence regions were conducted using Oligo 7 software (https://www.oligo.net/, accessed on 4 October 2025), focusing on those with ladder-shaped melting temperature curves, GC content varying between 40% and 80%, and verified good specificity through alignment (see Supplementary Materials Figure S1). The target sequences served as the basis for designing LMTIA primers specific to soybean, wheat, and peanut. This was accomplished with the Primer 3 Plus online tool (http://www.primer3plus.com, accessed on 4 October 2025). Initially, a set of forward primers (F) and reverse primers (R) was designed with a melting temperature ranging from 59 to 63 °C and a length of 18 to 24 nucleotides. LB were developed by initially creating a pair of nested primers and linking the sequences with “-tttt-”. Subsequently, Proofman probes were designed using the loop primer sequences, incorporating a quencher group at the 5′ end and a fluorescent reporter group at the 3′ end [24]. GenScript Biotechnology Co., Ltd. (Hefei, China) synthesized all primers. The details of these primers are provided in Table 1.

The LAMP primer sets were adopted directly from the Chinese Export Food Allergen Detection Standard [18,32,33]. Details of the primers are provided in Table 2.

### 2.3. DNA Extraction

Each sample was pulverized under liquid nitrogen, and 50 mg of powder was taken separately. DNA extraction from the samples was performed using a plant genomic DNA auto-extraction kit. A Qubit 4 fluorometer was used to quantify DNA concentration, and a NanoDrop One spectrophotometer was used for purity assessment. DNA specimens exhibiting both high concentration and a purity ratio of 1.8 to 2.0 were chosen [34]. Their completeness was validated by electrophoresis on a 1% agarose gel, and they were stored at −80 °C for future experiments.

### 2.4. Reaction System

Compared to the duplex system, the triplex assay was optimized by increasing the concentrations of Bst DNA polymerase, MgSO_4_, and premix buffer (5×). Furthermore, the primer and probe concentrations for all three targets were carefully balanced. These adjustments resulted in more comparable amplification curves across detection channels and reduced the risk of false-negative results [29].

The total reaction system of the single and triplex assays comprised 10 μL, which included 5× premix buffer, primers, probes, Bst pol, positive DNA, and DEPC-H_2_O. Subsequently, 20 μL of liquid paraffin was applied to the surface of the reaction solution to mitigate aerosol contamination. The 5× premix buffer contained Tris-HCl (pH 8.8 at 25 °C), KCl, ammonium sulfate, magnesium sulfate, Triton X-100, and dNTPs. Since the multiplex reaction involves an increased number of primers and probes, it requires larger amounts of substrates and enzymes. Therefore, the contents of the premix buffer, Bst polymerase, and MgSO_4_ were increased in the multiplex system. Amplification reactions were conducted using the Gentier 96E medical PCR analysis system (Tianlong Science and Technology, Xi’an, China). The configuration of this system was delineated in Table 3.

The LAMP reaction systems were adopted directly from the Chinese Export Food Allergen Detection Standard. The composition of this system is presented in Table 4. The Chinese Export Food Allergen Detection Standard [18,32,33] served as the direct source for the LAMP reaction systems. The makeup of this system is provided in Table 4.

### 2.5. Optimizing the Temperature of the Single-Plex System

Proofman-LMTIA reactions for each target (soybean, wheat, and peanut) were first performed to establish their respective optimal temperature ranges. To determine the optimal primer sets for the three allergens, preliminary experiments were conducted to screen the two primer pairs. The single reaction system outlined in Table 3 (right) was used genomic DNA of soybean, wheat, or peanut as positive controls, with DEPC-H_2_O-treated water as the negative control. Temperature optimization was performed for each allergen using the optimal primer sets. For the single reaction system described in Table 3, genomic DNA from soybean, wheat, or peanut served as positive controls. DEPC-H_2_O was used as the negative control. The optimal primer sets were then employed to optimize the temperature for each allergen. The temperature range tested for the wheat, soybean, and peanut allergens was 60, 61, 62, 63, 64, and 65 °C. Fluorescence signals were recorded at 30 s intervals for each of the 40 cycles. Each condition was tested with two replicates, and the whole experiment was carried out three times independently.

### 2.6. Temperature Optimization of the Triple Testing Method

Following the reaction system outlined in Table 3, positive controls consisted of soybean, wheat, and peanut genomes, while DEPC-H_2_O served as the negative control. Reaction temperatures were set between 60 and 63 °C. Each cycle lasted one minute, and fluorescence signals were captured at the end of each cycle for a total of 30 cycles. For every experiment, two parallel samples were prepared, and the entire study was carried out in triplicate.

### 2.7. Specificity Testing of the Triple Testing Method

The specificity of the triple system was evaluated using gDNA from rice, corn, walnut, sesame, cashew, and almond, which share a close phylogenetic relationship or are commonly encountered. The genome of soybean, wheat, or peanut (5 ng/μL) served as the positive control, whereas DNA from rice, walnut, sesame, cashew, and almond (5 ng/μL), in addition to DEPC-H_2_O, functioned as the negative control. One cycle was run per minute, and a fluorescence reading was taken each cycle; altogether, 30 cycles were completed. The reaction mix was incubated at the optimized temperature for 30 min. Two replicates were prepared for each experimental group, and the whole experiment was carried out independently three times.

### 2.8. Repeatability Testing of the Triple Detection Method

Genomic DNA from soybean, wheat, and peanut was mixed at a 1:1:1 ratio to prepare a mixed solution at 5 ng/µL, which served as the positive control. DEPC-treated water was used as the negative control. Using the optimal temperature and reaction system, triple rapid detection was carried out. One cycle was performed, and a fluorescence signal was captured at one-minute intervals, resulting in a total of 30 cycles. For each sample, eight parallel experiments were established. The experiment was performed in triplicate. The Ct value is a core indicator for determining positive or negative results in qualitative PCR detection. The lower the relative standard deviation (RSD) of Ct values, the better the stability and repeatability of the method. For nucleic acid amplification, an RSD of less than 5% is widely accepted as the criterion for good stability [35].

(1) $${\mathrm{RSD}}_{Ct}=\frac{{\mathrm{SD}}_{Ct}}{{Mean}_{Ct}}\times 100\%$$

(2) $${\mathrm{SD}}_{\mathrm{Ct}}=\sqrt{\frac{\sum_{i=1}^{n}{\left({\mathrm{Ct}}_{i}-\overset{-}{\mathrm{Ct}}\right)}^{2}}{n-1}}$$

### 2.9. Sensitivity Evaluation of the Triple Assay

To precisely establish the detection limit (LOD) of the Proofman-LMTIA method, a sensitivity test was performed using a fine concentration gradient [36]. We prepared dilutions of soybean, wheat, and peanut genomes. The concentrations used were 1 ng/μL, 500 pg/μL, 100 pg/μL, 50 pg/μL, 10 pg/μL, 5 pg/μL, and 1 pg/μL. The test samples comprised diluted genomes of soybean, wheat, and peanut at varying concentrations. The genomes of soybean, wheat, and peanut at a concentration of 1 ng/μL functioned as positive controls, while DEPC-H_2_O served as the negative control. Set each cycle to one minute, for a total of 40 cycles. The reaction mixture was incubated at the optimized temperature for 40 min. Two parallel samples were established for each experiment. The experiment was performed in triplicate.

### 2.10. Detection Limit Evaluation of the Triple Assay

The minimum detectable mass percentage of components using the triple Proofman-LMTIA system was assessed by pre-mixing soybean flour, wheat flour, and peanut flour with rice flour at mass percentages (*w*/*w*) of 1%, 3%, 5%, 8%, 10%, 15%, 25%, 50%, 75%, 90%, and 99%, respectively. DNA extraction was performed on different mixed samples utilizing the kit outlined in Section 2.3. The DNA obtained from the mixes served as the test sample. The gDNA from soybean, wheat, and peanut was utilized as positive controls. The gDNA of rice and DEPC-H_2_O was employed as the negative control. One cycle was performed, and one fluorescence signal was recorded every minute, with a total of 40 reaction cycles established, and amplification was conducted for 40 min at the optimized temperature. Two parallel samples were established for each experiment. The experiment was conducted in duplicate.

### 2.11. Application of the Triple Rapid Detection Method

Ten processed food products containing soybean, wheat, and peanut were purchased from the local market. These products included peanut butter, nut bread, oatmeal, fermented bean curd, yellow soybean pastes, soy sauce, wheat flour, soda crackers, instant ramen, and whole wheat bread. The specific operations for DNA extraction were carried out according to the method described in Section 2.3. For actual sample analysis, the established Proofman-LMTIA triple rapid testing method was applied. Genomic DNA extracted from soybean, wheat, and peanut was taken as the positive control, and DEPC-treated water served as the negative control. Set each cycle to one minute, for a total of 40 cycles The reaction occurred for 40 min at the optimal temperature. Two parallel groups were created for each experiment. The experiment was performed in triplicate.

### 2.12. LAMP Method Sensitivity Testing

According to the LAMP procedure described in Table 4, genomic DNA from wheat, soybean, and peanut was serially diluted to respective concentrations of 1 ng/μL, 500 pg/μL, 100 pg/μL, 50 pg/μL, 10 pg/μL, and 1 pg/μL. We used these diluted genomes as test samples. To evaluate the sensitivity of the single LAMP assays, the positive control was a mixture of wheat, soybean, and peanut genomic DNA, while the negative control was DEPC-treated water. The reactions were performed at the temperatures prescribed in the standard method: 63 °C for wheat and soybean, and 65 °C for peanut, for a duration of 40 min [18,32,33]. All experiments were conducted with two parallel replicates per sample and were repeated twice.

## 3. Results

### 3.1. Optimizing the Temperature of the Single-Plex System

Detailed results of primer screening and the remaining temperature optimization are provided in the Supplementary Materials Figures S2 and S3. As shown in Figure S3A–F, the single reaction systems for peanuts and soybeans were performed at 60–65 °C. At all six temperatures, the DNA templates from peanut and soybean exhibited positive amplification, whereas DEPC-H_2_O showed no amplification. At a reaction temperature of 61 °C, the amplification efficacy of the soybean DNA template and the peanut DNA template was maximized, demonstrating enhanced fluorescence intensity and greater stability. The peak amplification efficiency for wheat was recorded at 62 °C. The optimized reaction temperature for peanut and soybean Single LMTIA was 61 °C, while for wheat it was 62 °C.

### 3.2. Temperature Optimization of the Triple Testing Method

**Figure 1:**
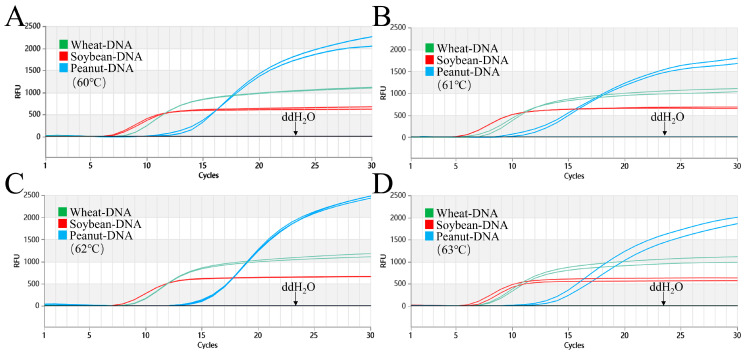
Optimization of triple Proofman-LMTIA reaction temperature for wheat, soybean, and peanut. (**A**–**D**): Amplification graphs of the triple Proofman-LMTIA reaction system for wheat, soybean, and peanut at temperatures of 60 °C, 61 °C, 62 °C, and 63 °C, respectively. Positive controls: wheat, soybean, and peanut gDNA; negative controls: DEPC-H_2_O.

Based on the results from the single assays, the triple reaction was evaluated at 60–63 °C. As shown in Figure 1, at all four temperatures, the positive DNA exhibited a marked signal enhancement, with no amplification observed in the negative control (DEPC-H_2_O). Although wheat and peanut each exhibited optimal amplification efficiency at 60 °C and 62 °C, respectively, a unified temperature was required to enable the simultaneous detection of all three allergens in a triplex format. At 61 °C, soybean exhibited amplification as early as cycle 5 ($\bar{Ct}$ value of 6.5), while wheat and peanut showed $\bar{Ct}$ values of 8.5 and 12, respectively, all maintaining high amplification efficiency. Therefore, 61 °C was selected as the optimal temperature for the triplex reaction.

### 3.3. Specificity of the Triple Reaction

**Figure 2:**
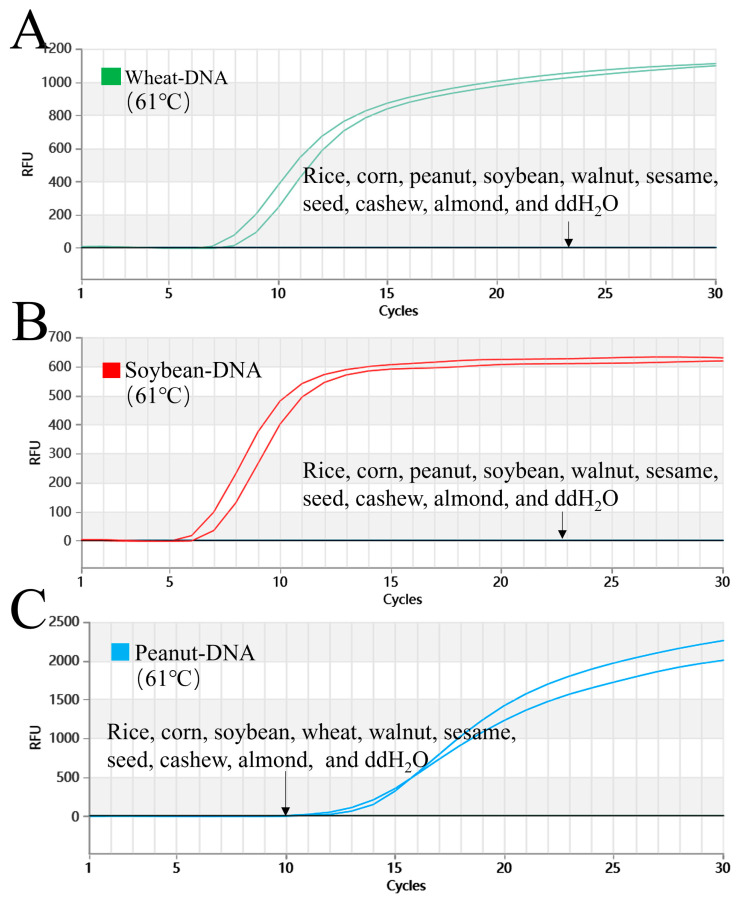
Specificity assessment of the triple Proofman-LMTIA assay at 61 °C for wheat, soybean, and peanut. (**A**–**C**): Amplification graphs of the triple Proofman-LMTIA reaction system at 61 °C for the testing of wheat, soybean, and peanut specificity. Positive controls: gDNA of wheat, soybean, or peanut; negative controls: gDNA of rice, corn, soybean, peanut, wheat, walnut, sesame, cashew, almond, and DEPC-H_2_O.

As shown in Figure 2, the triple Proofman-LMTIA method demonstrated high specificity. No amplification was detected in non-target species, including rice, corn, walnut, sesame, cashew, and almond, or in the DEPC-H_2_O (negative control). In contrast, the positive controls for wheat, soybean, and peanut all showed strong amplification. These results confirmed the high specificity of the established triple Proofman-LMTIA reaction system.

### 3.4. Repeatability of the Triple Reaction

As shown in Figure S4, at the optimal temperature of 61 °C, all positive DNA underwent amplification, whereas DEPC-H_2_O exhibited no amplification. The Ct values for the wheat allergen ranged from 9.3 to 9.8 (RSD of 1.2%); for the soybean allergen, the Ct values ranged from 10.8 to 11.9 (RSD of 2.6%); and for the peanut allergen, the Ct values ranged from 9.8 to 10.3 (RSD of 1.5%). The variation among replicate samples for the three allergens was extremely low (RSD < 5%, with n = 8), indicating strong repeatability.

### 3.5. Sensitivity of the Triple Reaction

**Figure 3:**
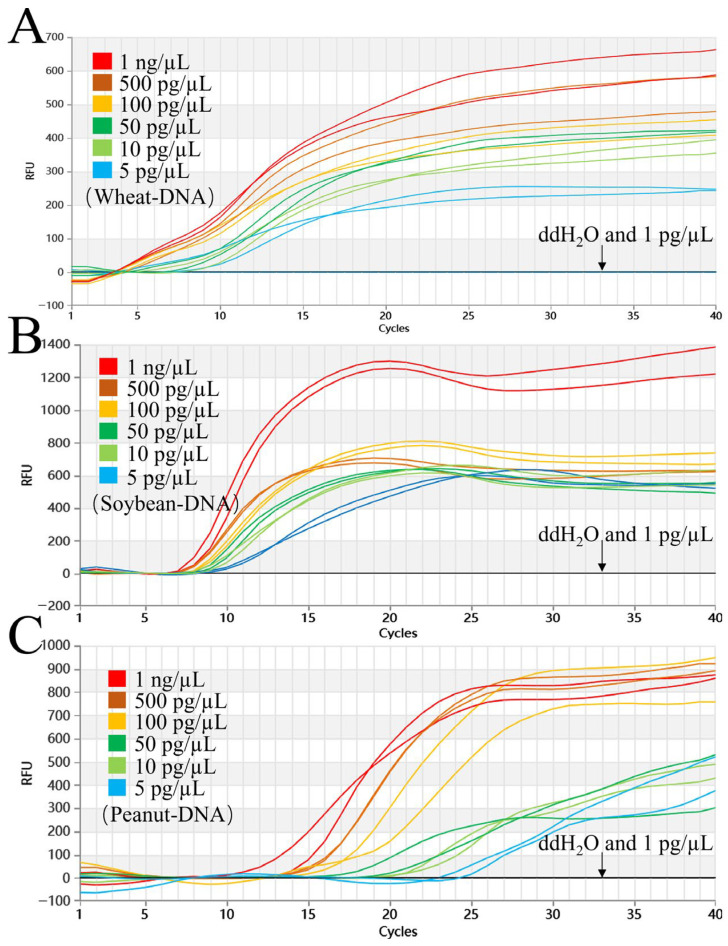
Sensitivity of the triple Proofman-LMTIA assay at 61 °C for wheat, soybean, and peanut. (**A**–**C**): Amplification graphs of the triple Proofman-LMTIA reaction system at 61 °C for wheat, soybean, and peanut sensitivity. The gDNA of wheat, soybean, and peanut was diluted at 1 ng/µL, 500 pg/µL, 100 pg/µL, 50 pg/µL, 10 pg/µL, 5 pg/µL, and 1 pg/µL. These served as the test groups. Positive controls: gDNA of wheat, soybean, and peanut; negative controls: DEPC-H_2_O.

When tested at 61 °C, the triple system showed clear amplification signals for wheat, peanut, and soybean DNA across concentrations of 1 ng/μL, 500 pg/μL, 100 pg/μL, 50 pg/μL, 10 pg/μL, and 5 pg/μL. Neither the negative control nor the 1 pg/μL sample produced any amplification (Figure 3). With this system, we successfully detected the three target genomes down to 5 pg/μL.

### 3.6. Detection Limit of the Triple Reaction

**Figure 4:**
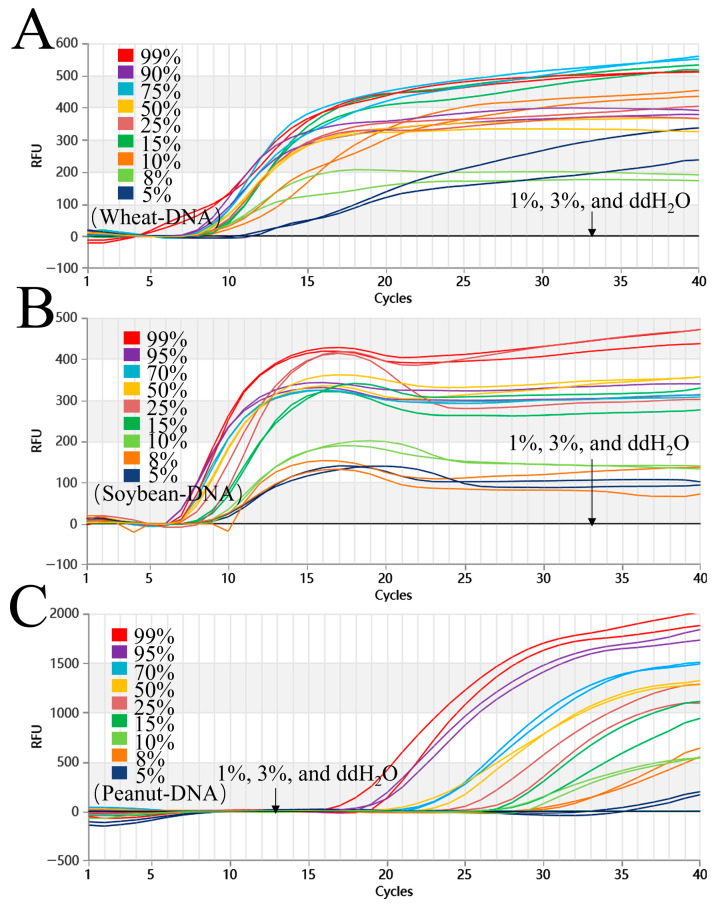
Detection limit assessment of the triple Proofman-LMTIA assay at 61 °C for wheat, soybean, and peanut. (**A**–**C**): Amplification graphs of the triple Proofman-LMTIA reaction system at 61 °C for wheat, soybean, and peanut detection limit. Wheat, soybean and peanut were blended with rice at varying mass fractions of 99%, 90%, 75%, 50%, 25%, 15%, 10%, 8%, 5%, 3%, and 1%, respectively. These served as the test groups. Positive controls: gDNA of wheat, soybean, and peanut; negative controls: DEPC-H_2_O.

The results indicated that amplification curves were detected for wheat, peanut, and soybean mass ratios at 99%, 90%, 75%, 50%, 25%, 15%, 10%, 8%, and 5% (*w*/*w*) (Figure 4). However, no amplification was identified in DEPC-H_2_O or in the 3% and 1% samples. The triple reaction system had a limit of detection of 5% (*w*/*w*).

### 3.7. Detection of Actual Samples

To assess the real-world use of the triple detection system, 10 commercially available products containing wheat, peanut, or soybean ingredients were analyzed. As summarized in Table 5, no amplification signals were observed in any negative controls, indicating the absence of contamination throughout the experimental procedure. Two allergens were simultaneously detected in samples 1 and 3; soybean was identified in samples 4–6, wheat in samples 7–10, and peanut in sample 2. The detection outcomes matched perfectly with the allergen components listed on the product labels. For further details, see Figure S5 in the Supplementary Materials.

### 3.8. LAMP Method Sensitivity Testing

**Figure 5:**
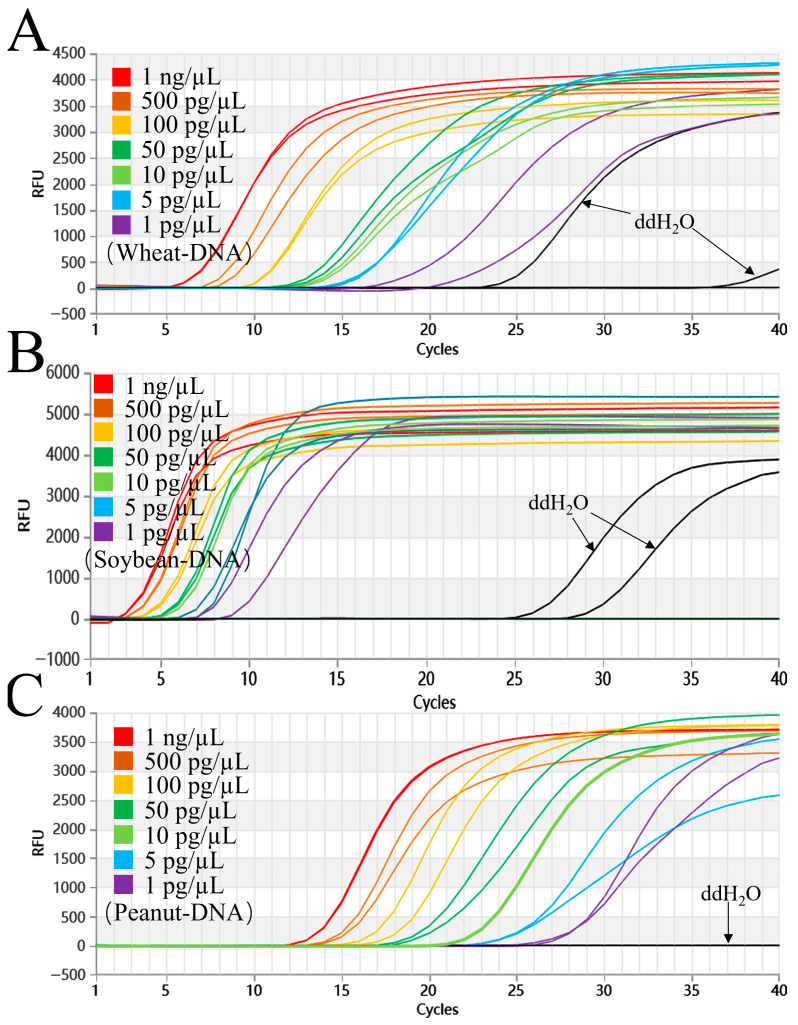
Sensitivity of the LAMP assay for wheat, soybean, and peanut. (**A**–**C**): Amplification graphs of the LAMP reaction system for wheat, soybean, and peanut sensitivity. The gDNA of wheat, soybean, and peanut was diluted at 1 ng/µL, 500 pg/µL, 100 pg/µL, 50 pg/µL,10 pg/µL, 5 pg/µL, and 1 pg/µL. These served as the test groups. Positive controls: gDNA of wheat, soybean and peanut; negative controls: DEPC-H_2_O.

As shown in Figure 5, the LAMP assay demonstrated excellent sensitivity, successfully detecting wheat, soybean, and peanut genomic DNA across a concentration range of 1 ng/μL, 500 pg/μL, 100 pg/μL, 50 pg/μL, 10 pg/μL, and 1 pg/μL. However, the DEPC-H_2_O repeatedly showed non-specific amplification across several independent experiments. While the sensitivity of the LAMP method for wheat, soybean, and peanut DNA reached 1 pg/μL, the appearance of false positives may substantially compromise the reliability of the detection.

## 4. Discussion

Wheat, soybean, and peanut are significant agricultural commodities and rank among the nine most prevalent food allergens [6]. The global incidence of food allergies has increased significantly, posing a critical public health issue. Statistics reveal that roughly 5% of adults and 8% of children are affected, with prevalence rates rising annually [14]. In the U.S., the incidence of pediatric peanut allergy has increased 3.5-fold in the last decade [37]. Although food packaging typically lists allergen information, cross-contamination during production, processing, storage, transportation, or equipment cleaning can lead to discrepancies between actual and labelled composition. Moreover, inadequate allergen management in food service and educational settings intensifies related public health risks.

Significant research endeavors have focused on developing multiplex detection platforms for allergens. Ref. [38] developed a duplex ELISA for detecting the primary peanut allergens Ara h 2 and Ara h 6 in human milk, achieving limits of detection (LOD) of 1.3 ng/mL and 0.7 ng/mL, respectively. Ref. [39] developed an xMAP food allergen detection assay that can concurrently identify 15 food allergens with high sensitivity and reliability. Reference [40] presented an LC-MS/MS methodology for the detection of allergens from egg white, skimmed milk, peanuts, soybeans, and tree nuts, with adequate recovery (60–119%), repeatability (RSD < 20%), and sensitivity (LOD = 10 ppm in biscuits and bread). Ref. [41] employed multiplex PCR, achieving a limit of detection of approximately 0.08 ng for fruit allergens such as tomato, apple, peach, and kiwi. Furthermore, ref. [42] developed the microfluidic LAMP assay with a colorimetric readout for peanut, sesame, and soybean, achieving a detection limit (LOD) of 0.4 ng/μL.

Although the aforementioned methods enable multiplex detection, each presents significant limitations in practical applications. Immunoassays such as ELISA and xMAP offer advantages including operational simplicity, field deployability, and high throughput; however, their performance is heavily dependent on antibody quality. Protein denaturation during food processing can severely compromise detection accuracy, and cross-reactivity among structurally similar proteins frequently leads to false-positive results [36]. Mass spectrometry, despite its exceptional specificity and sensitivity, relies on costly apparatus and specialist knowledge, limiting its application to laboratory environments. The microsphere-based xMAP technology, while possessing exceptional multiplexing capabilities, requires costly flow cytometers, resulting in high detection costs. Compared to proteins, DNA exhibits superior stability throughout food processing. Although multiplex PCR and DNA microarrays enable high-throughput detection, they are limited by their requirement for thermal cyclers, extended run times, and, in the case of microarrays, the need for complex data analysis and dedicated scanners. Loop-mediated isothermal amplification (LAMP) technology, despite its isothermal nature enabling on-site detection when combined with visual analysis, is still vulnerable to false-positive outcomes due to non-specific amplification.

In contrast, the Proofman-LMTIA method developed here strikes a better balance across these factors. By incorporating lyophilized microsphere technology, this method requires only a portable isothermal amplification instrument to complete on-site rapid detection within 40 min. Its reagent costs are comparable to those of standard LAMP or PCR assays, yet the operational workflow is more streamlined and effectively avoids the false-positive issues commonly encountered with LAMP methods. Consequently, Proofman-LMTIA significantly enhances detection accuracy while maintaining the advantages of rapidity, convenience, and low cost, thereby offering a more reliable solution for on-site screening of food allergens.

In this study, the LAMP primer and system referenced from the standard method, combined with the use of a fluorescent dye, demonstrated exceptionally high sensitivity and stability, with a limit of detection (LOD) as low as 1 pg/μL. However, a notable limitation of this technique was observed: even after eliminating operational errors and conducting multiple repeated experiments, non-specific amplification could still be detected. Meanwhile, the LMTIA technology employed in this research targets a shorter fragment derived from the LAMP target sequence, and no non-specific amplification was observed in the LMTIA system. This indicates that the LAMP method described in the standard carries a risk of false positives, likely due to non-specific amplification induced by primer-dimer formation [43].

To overcome these limitations, we developed a triple Proofman-LMTIA assay. This nucleic acid-based technique is rapid (40 min), functions isothermally without the necessity for thermal cycling or gel electrophoresis, and eliminates the need for advanced instrumentation. The assay exhibited high specificity when assessed against DNA from phylogenetically related species or commonly consumed foods. The sensitivity for wheat, peanut, and soybean DNA was 5 pg/μL, with a detection limit in food matrices of 5% (*w*/*w*). The gene segments of *Lectin*, *GAG56D*, and *Ara h 2.01* functioned as precise targets for soybean, wheat, and peanut allergens, respectively, exhibiting remarkable specificity. Validation with ten commercial food products demonstrated full concordance with label declarations, effectively identifying both single and multi-allergen contamination (Table 5), and affirming assay reliability in complex food matrices. Although the standard LAMP method achieves an LOD as low as 1 pg/μL, it is associated with the risk of false-positive results in our hands under the described conditions. Prior to this study, we also optimized the single Proofman-LMTIA assays for the three allergens. Each assay exhibited high specificity with an amplification time of only 20 min, achieving sensitivities of 1 pg/μL for wheat, 10 fg/μL for soybean, and 100 fg/μL for peanut (Figure S6A–C). Notably, no non-specific amplification was observed in either the single-plex or triplex detection formats, effectively eliminating false-positive results. While the single-plex assay demonstrated extremely high sensitivity and a shorter amplification time, the triplex assay maintained excellent sensitivity (5 pg/μL per target) while enabling simultaneous detection of the three allergens, thereby significantly improving detection efficiency. In terms of analytical sensitivity, the LAMP-based microfluidic method established by Yuan et al. [42] for peanut, sesame, and soybean achieved a sensitivity of 0.4 ng/μL, whereas the LAMP assay developed by Mao et al. [44] for pistachio and cashew reached a sensitivity of 6.4 pg/μL. The triple Proofman-LMTIA system established in this study achieved a sensitivity of 5 pg/μL for soybean, wheat, and peanut allergens, which is sufficient for the detection of allergenic species in routine food samples. For applications requiring trace-level detection, the single Proofman-LMTIA format can be employed to achieve even higher sensitivity. The advantages and disadvantages of common food allergen detection methods are shown in Table 6.

This study has several limitations that should be acknowledged. First, nucleic acid detection is an indirect approach, whereas the primary trigger of allergic reactions is allergenic proteins; therefore, the detection results may not fully align with the actual allergenic protein content. Second, the scope of detection targets is relatively narrow, currently covering only soybean, wheat, and peanut allergens. Ongoing research is expanding to include milk and selected seafood allergens. Third, for allergens with high protein content but extremely low DNA content, such as egg, nucleic acid-based methods are less applicable, and protein-based detection methods are more suitable. Fourth, the current method is limited to qualitative detection and has not yet achieved precise quantification of allergenic components. Fifth, in extremely complex matrices, such as deeply processed foods with high salt, sugar, or fat content, the detection performance of this method still requires further improvement. Future studies will focus on addressing these limitations.

Regarding technological integration and innovation, the LMTIA method can be combined with visual analysis and microfluidic chip technology to further simplify result interpretation, eliminating the dependence on dedicated fluorescence detection instruments and enabling intuitive visual observation of positive and negative reactions. This advancement allows result interpretation without the need for specialized personnel, significantly lowering the detection threshold and making the method more suitable for on-site rapid screening. Furthermore, DNA extraction serves as the foundation of molecular biology research, and the quality of extracted DNA critically influences the accuracy of nucleic acid detection. Although rapid nucleic acid detection technologies have matured considerably, achieving rapid and simple high-quality DNA extraction remains a major challenge for on-site testing applications.

## 5. Conclusions

In summary, a novel triple Proofman-LMTIA assay was developed in this work for the concurrent and fast detection of three allergens: wheat, peanut, and soybean. Compared with the existing LAMP standard method, this approach achieves multiplex detection with high sensitivity, specificity, and stability, requiring only 40 min to complete the assay, achieving detection limits of 5 pg/μL and 5% (*w*/*w*), while effectively eliminating false-positive results. Its isothermal nature, short detection time, and operation without relying on sophisticated instrumentation make it particularly suitable for on-site applications. Successful validation using commercial food samples further confirms the reliability and practical utility of this technology for accurate allergen detection in complex matrices, demonstrating its significant potential in food safety monitoring and industrial self-testing. While the method shows promise, further validation is required for trace-level allergen detection, broader food matrices, and real-world regulatory deployment.

## Figures and Tables

**Table 1:** The triplex Proofman-LMTIA primers and fluorescent probes.

Primers and Probes	Sequence (5′ to 3′)
WH-F	CTTTGTGGCCAGATTTTTTTGGTGTCATCCCTCTGGTCA
WH-B	TCTGGCCACAAAGCGTTTTCTGTGCTAGTTGTTGGCAGC
WH-LB	ATTGCCAAGTGATGC
SO-F	TTTGTGTCAGGGGCTTTTTTCGCCGCTTCCTTCAACTT
SO-B	CCCCTGACACAAAATTTTTTGGTGCGAGAAAGAAGGCA
SO-LB	AGGCTTGCAGATGGGC
PE-F	CCGTTCATATGAATCTTTTCCCTGCGAGCAACATCTCAT
PE-B	ATATGAACGGGACTTTTGATCCTGACTAGGGCTGTACGG
PE-LB	CTCGTGACGAGGATTC
WH-Probe1	BHQ2-ATTGCCAAGTGATGT-JOE
SO-Probe2	BHQ2-AGGCTTGCAGATGA-CY5
PE-Probe3	BHQ2-GTGACGAGGATTT-FAM

F: forward outer primer; B: backward outer primer; LB: loop backward primer. WH: wheat (*Triticum aestivum*). SO: soybean (*Glycine max*). PE: peanut (*Arachis hypogaea*). Typically, probes are labeled with a fluorescent group at the 3′ end and a quencher group at the 5′ end.

**Table 2:** LAMP primers.

Primers	Sequence (5′ to 3′)
WH-F	CAGCAACCACAACAACAATT
WH-B	TAGTTGTTGGCAGCATTGT
WH-FIP	TTCTTGCATGGGTTCACCTGTTCAACCGCAACAATCATTCC
WH-BIP	AACCTGTGTCACTGGTGTCATCATCACTTGGCAATCGCTT
WH-FLP	TAGAGATGGCTGAATGAACGG
WH-BLP	CCTCTGGTCAATGATCTGGC
SO-F	AAGAAACCGGTAGCGTTG
SO-B	AAGTGTCAAACTCAACAGC
SO-FIP	AAGCCCATCTGCAAGCCTTTGCCGCTTCCTTCAACTTC
SO-BIP	TTCTCGCACCAATTGACACTAAGACCAGACTCGTTTTCGTTG
SO-FLP	GTGTCAGGGGCATAGAAGGTG
SO-BLP	AACACATGCAGGTTATCTTGGTCT
PE-F	CCCTGCGAGCAACATCTC
PE-B	TGCACCTTTGGTTGTTCTCA
PE-FIP	ATCCTGACTAGGGCTGTACGGGTGCAGAAGATCCAACGTGAC
PE-BIP	GTCCATATGATCGGAGAGGCGCCGTTCAGCTCATTGCAACAC

F: forward outer primer; B: backward outer primer; FIP: forward inner primer; BIP: backward inner primer; FLP: forward loop primer; BLP: backward loop primer. WH: wheat (*Triticum aestivum*). SO: soybean (*Glycine max*). PE: peanut (*Arachis hypogaea*).

**Table 3:** Single (right) and triplex (left) Proofman-LMTIA reaction system (10 µL).

Triple Reaction System		Single Reaction System	
Composition	One Tube (μL)	Composition	One Tube (μL)
LMTIA premix buffer (5×)	3	LMTIA premix buffer (5×)	2
Bst Polymerase (100 U/µL)	0.6	Bst Polymerase (100 U/µL)	0.4
MgSO4 (100 mM)	0.15	WH/SO/PE-F (100 μM)	0.16
WH-SO-PE-F (100 μM)	0.16 + 0.16 + 0.16	WH/SO/PE-B (100 μM)	0.16
WH-SO-PE-B (100 μM)	0.16 + 0.16 + 0.16	WH/SO/PE-LB (100 μM)	0.04
WH-SO-PE-LB (100 μM)	0.04 + 0.04 + 0.04	WH/SO/PE-probe (10 μM)	0.4/0.1/0.2
WH-SO-PE-probe (10 μM)	0.4 + 0.1 + 0.2	P-DNA (5 ng/μL)	2
P-DNA (5 ng/μL)	2	DEPC-H_2_O	Add to 10 µL
DEPC-H_2_O	Add to 10 µL		

WH: wheat (*Triticum aestivum*); SO: soybean (*Glycine max*); PE: peanut (*Arachis hypogaea*); P-DNA: positive control DNA.

**Table 4:** LAMP reaction system (25 µL).

Composition	One Tube (μL)
ThermoPol Buffer (10×)	2.5
WH/SO/PE-F3 (5 μM)	1
WH/SO/PE-B3 (5 μM)	1
WH/SO/PE-FIP (40 μM)	1
WH/SO/PE-BIP (40 μM)	1
WH/SO-FLP (10 μM)	1
WH/SO-BLP (10 μM)	1
dNTPs (10 mMM)	5
Betaine (5 mol/L)	4
MgSO4 (100 mM)	0.5
Bst DNA polymerase (8 U/μL)	1
DNA template (10 ng/μL)	2
SYTO 9 (50 μM)	1
DEPC-H_2_O	Add to 25 µL

WH: wheat (*Triticum aestivum*). SO: soybean (*Glycine max*). PE: peanut (*Arachis hypogaea*).

**Table 5:** Detection results of commercial wheat, soybean, and peanut allergen components.

No	Samples	Labeling Ingredients	Wheat	Soybean	Peanut
1	Nut bread	Wheat, peanut	+	−	+
2	Peanut butter	Peanut	−	−	+
3	Oatmeal	Soybean, Wheat	+	+	−
4	Fermented bean curd	Soybean	−	+	−
5	Yellow soybean pastes	Soybean	−	+	−
6	Soy sauce	Soybean	−	+	−
7	Wheat flour	Wheat	+	−	−
8	Soda crackers	Wheat	+	−	−
9	Instant ramen	Wheat	+	−	−
10	Whole wheat bread	Wheat	+	−	−

**Table 6:** Comparison of advantages and disadvantages of common food allergen detection methods.

Detection Method	Main Advantages	Main Disadvantages
ELISA	Mature technology, high sensitivity, strong specificity, quantitative capability, relatively moderate reagent cost	Susceptible to high-temperature and high-pressure processing, leading to false negatives; cumbersome operation, time-consuming, unsuitable for on-site rapid detection
LFIA	Simple and rapid operation, no complex instrumentation required, low cost, suitable for preliminary screening, good portability	Relatively low sensitivity, prone to matrix interference, poor quantitative capability; also susceptible to protein denaturation during processing, leading to false negatives
Mass Spectrometry (MS)	High detection accuracy, precise identification of allergenic proteins, strong anti-interference capability, wide application range	Expensive instrumentation, complex operation, high professional expertise required, high detection cost, unsuitable for on-site detection
Conventional PCR	Simple operation, relatively low reagent cost, rapid amplification of allergen genes, moderate technical requirements	Requires electrophoresis for result interpretation, cannot be accurately quantified; open-tube operation prone to contamination, relies on thermal cycler, poor portability
Real-time (qPCR)	High sensitivity, accurate quantification via standard curve, good specificity, reliable results, mature technology	Relies on precise thermal cycler, poor portability, unsuitable for on-site detection
Digital PCR (dPCR)	High sensitivity, absolute quantification without standard curve, strong anti-interference capability	Expensive instrumentation, low throughput, cumbersome droplet preparation, time-consuming, difficult for widespread application
LAMP	High sensitivity, no need for precise thermal cycler, relatively simple operation, faster amplification than conventional PCR	Complex primer design; high primer concentration and aerosol contamination prone to false-positive amplification; cannot achieve quantification
Proofman-LMTIA	High detection efficiency (20 min for single-plex, 30–40 min for multiplex); strong specificity (precise primer and probe design, no non-specific amplification, no cross-reactivity); simple instrumentation, easy operation, suitable for on-site detection; controllable cost, easy to promote; multiplex detection capability; strong anti-interference capability	Cannot achieve quantification, only qualitative analysis; primer design, although simpler than LAMP, still requires professional knowledge and software, posing certain difficulty; not suitable for species with very low DNA content (e.g., eggs)

## Data Availability

The original contributions presented in this study are included in the article/Supplementary Material. Further inquiries can be directed to the corresponding authors.

## Supplementary Materials

The following supporting information can be downloaded at: https://www.mdpi.com/article/10.3390/foods15081340/s1, Figure S1. Ladder-shape melting temperature profiles of the soybean lectin gene and wheat GAG56D gene sequences. Figure S2. Optimisation of wheat, soybean and peanut allergen primer reactions. Figure S3. Optimization of single Proofman-LMTIA reaction temperature for wheat, soybean, and peanut. Figure S4. Repeatability assessment of the triple Proofman-LMTIA assay at 61 °C for wheat, soybean, and peanut. Figure S5. Market sample data figure. Figure S6. The sensitivity of the single-plex Proofman-LMTIA assays for soybean, wheat, and peanut.
