# Supplementary material for: Triplex Proofman-LMTIA: A Rapid, Specific, and Sensitive Assay for Detecting Wheat, Peanut, and Soybean Allergens in Foods
Source: Foods. 2026 Apr 12;15(8):1340. doi: 10.3390/foods15081340 (PMC13115237; doi:10.3390/foods15081340)
Supplement: Supplementary file 1 [file foods-15-01340-s001.zip › foods-4222892-supplementary.pdf]

Supplementary Material

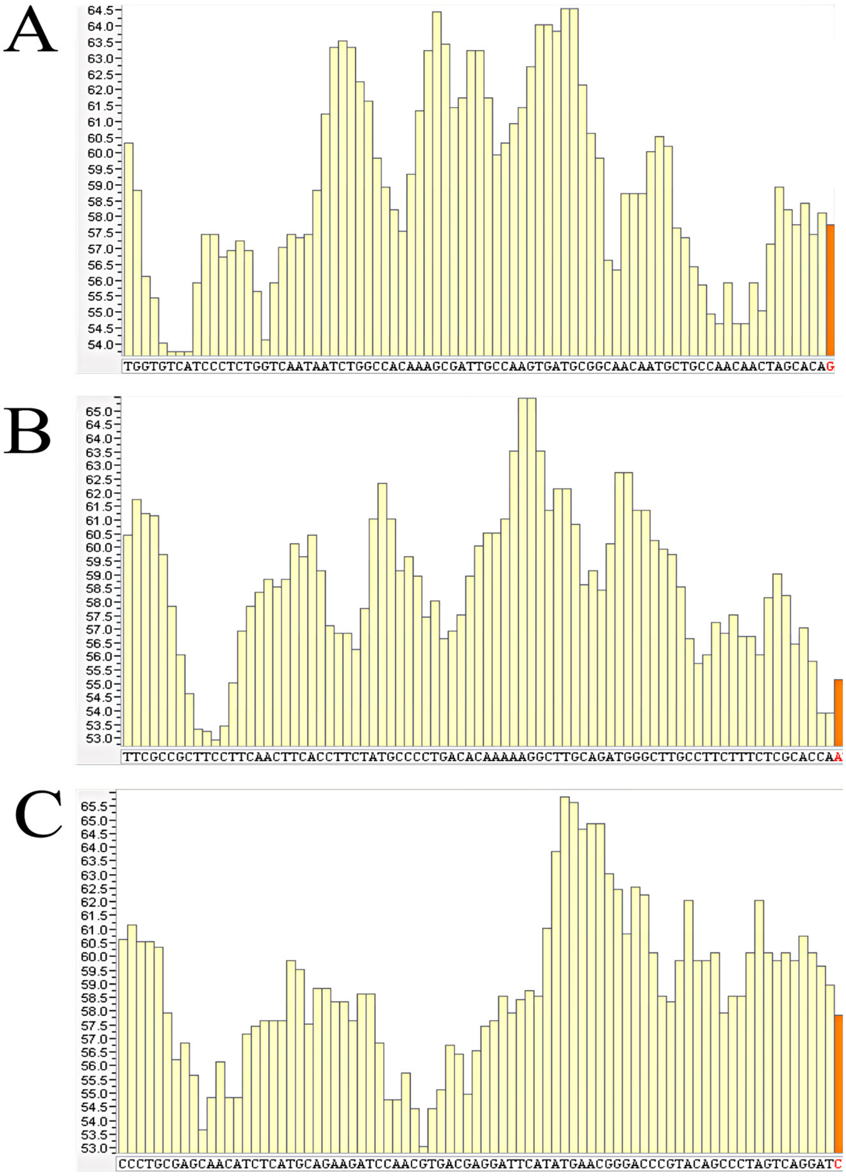

Figure S1. Ladder-shape melting temperature profiles of the soybean lectin gene and wheat GAG56D gene sequences. A: Melting temperature profile of the wheat GAG56D gene sequence; B: Melting temperature profile of the soybean lectin gene sequence; C: Melting temperature profile of the peanut Ara h 2.01 gene sequence.

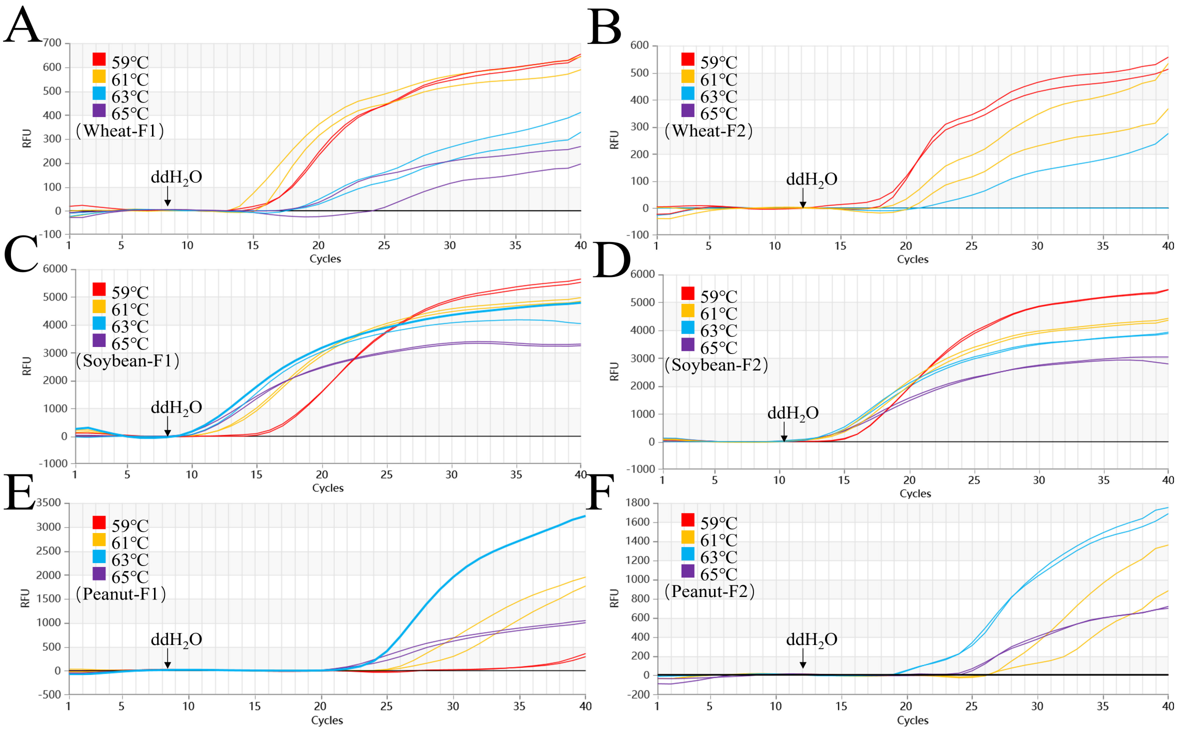

Figure. S2. Optimisation of wheat, soybean and peanut allergen primer reactions. A-B: Amplification curves for two primer reaction systems of wheat at 59, 61, 63 and 65°C. C-D: Amplification curves for soybean using two primer sets at 59, 61, 63 and 65°C. E-F: Amplification curves for peanut using two primer sets at 59, 61, 63 and 65°C. Positive controls: gDNA from wheat, soybean and peanut; Negative control: DEPC-H_2O.

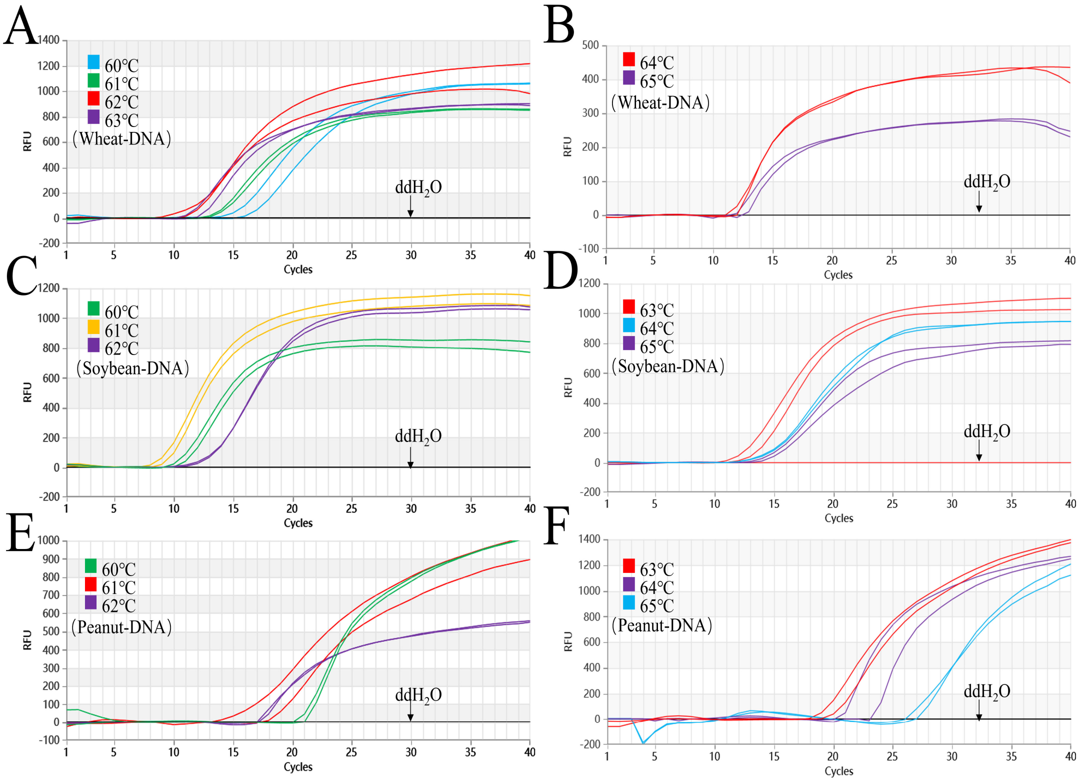

Figure. S3. Optimization of single Proofman-LMTIA reaction temperature for wheat, soybean, and peanut. A-B: Proofman-LMTIA reaction system amplification graph for wheat at 60, 61, 62, 63, 64, and 65°C. C-D: Proofman-LMTIA reaction system amplification graph for soybean at 60, 61, 62, 63, 64, and 65°C. E-F: Proofman-LMTIA reaction system amplification graph for peanut at 660, 61, 62, 63, 64, and 65°C. Positive controls: wheat, soybean, and peanut. gDNA; negative controls: DEPC-H_2O.

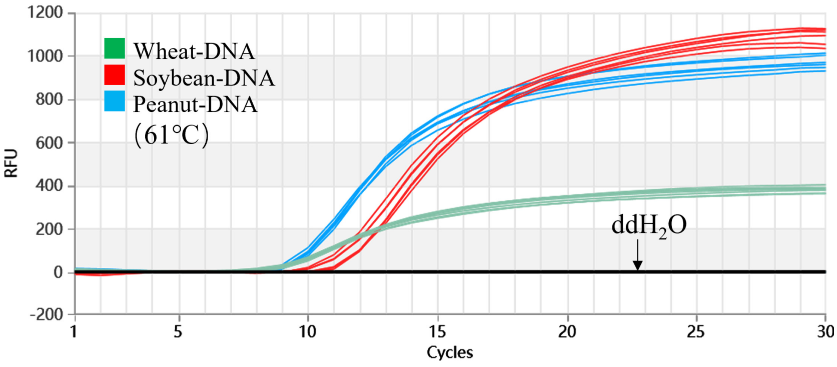

Figure. S4. Repeatability assessment of the triple Proofman-LMTIA assay at 61℃ for wheat, soybean, and peanut. Positive controls: gDNA of wheat, soybean, and peanut (1:1:1); negative controls: DEPC-H_2O.

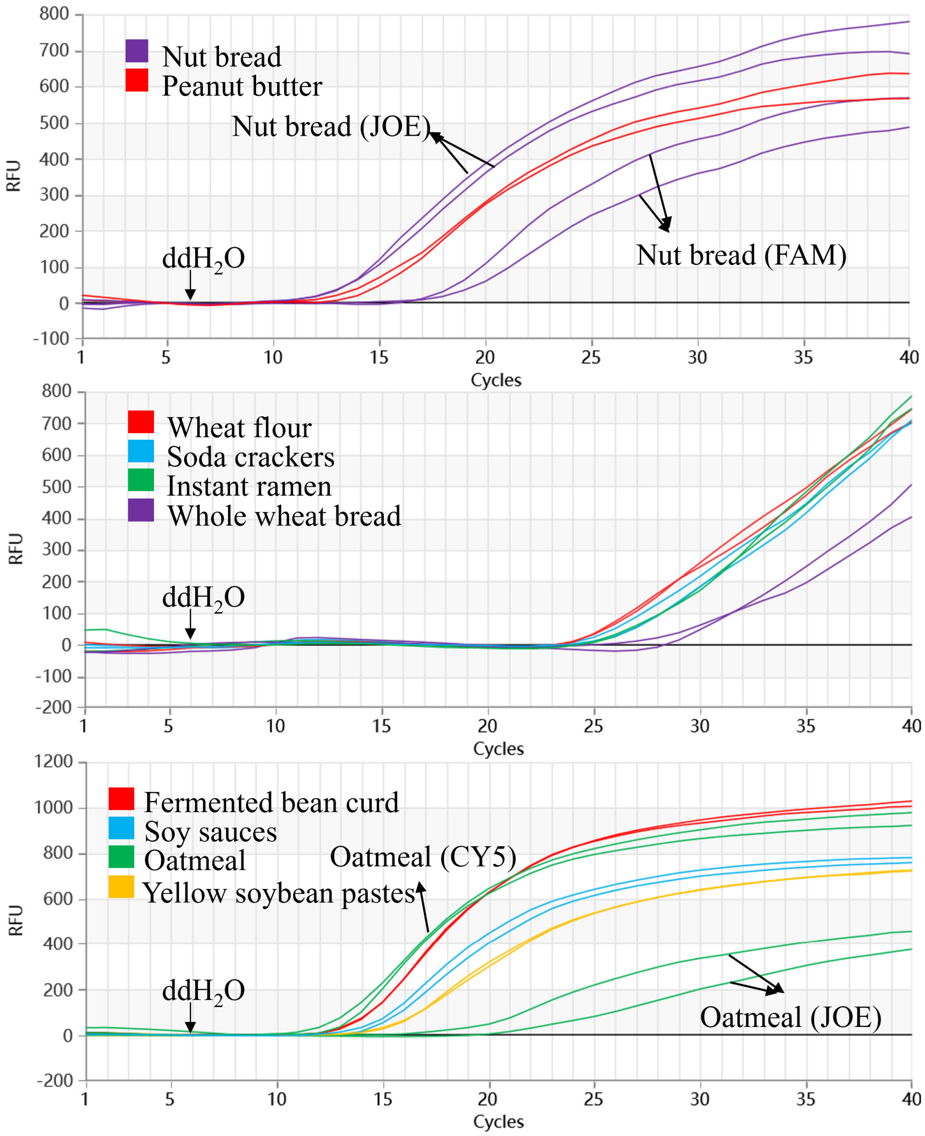

Figure. S5. Market sample data figure

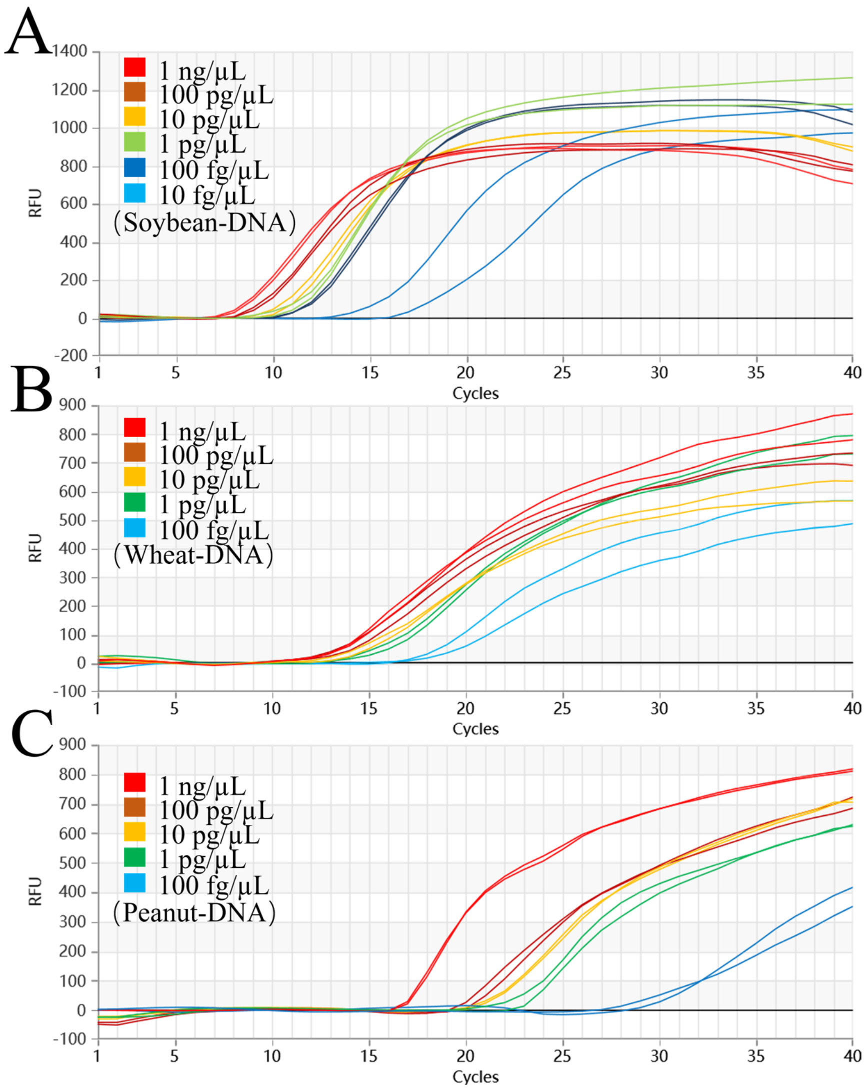

Figure. S6. The sensitivity of the single-plex Proofman-LMTIA assays for soybean, wheat, and peanut
